# Novel Ameloblastin Variants, Contrasting Amelogenesis Imperfecta Phenotypes

**DOI:** 10.1177/00220345231203694

**Published:** 2023-12-06

**Authors:** U. Hany, C.M. Watson, L. Liu, G. Nikolopoulos, C.E.L. Smith, J.A. Poulter, C.J. Brown, A. Patel, H.D. Rodd, R. Balmer, A. Harfoush, M. Al-Jawad, C.F. Inglehearn, A.J. Mighell

**Affiliations:** 1 Leeds Institute of Medical Research, University of Leeds, St. James’s University Hospital, Leeds, UK; 2 North East and Yorkshire Genomic Laboratory Hub, Central Lab, St. James’s University Hospital, Leeds, UK; 3 School of Dentistry, Clarendon Way, University of Leeds, Leeds, UK; 4 Birmingham Dental Hospital, Mill Pool Way, Edgbaston, Birmingham, UK; 5 LCRN West Midlands Core Team, NIHR Clinical Research Network (CRN), Birmingham Research Park (West Wing), Edgbaston, Birmingham, UK; 6 Academic Unit of Oral Health Dentistry and Society, School of Clinical Dentistry, University of Sheffield, Sheffield, S Yorks, UK

**Keywords:** AMBN, amelogenesis, dental enamelhypoplastic AI, founder effect, X-ray microtomography

## Abstract

Amelogenesis imperfecta (AI) comprises a group of rare, inherited disorders with abnormal enamel formation. Ameloblastin (AMBN), the second most abundant enamel matrix protein (EMP), plays a critical role in amelogenesis. Pathogenic biallelic loss-of-function *AMBN* variants are known to cause recessive hypoplastic AI. A report of a family with dominant hypoplastic AI attributed to AMBN missense change p.Pro357Ser, together with data from animal models, suggests that the consequences of *AMBN* variants in human AI remain incompletely characterized. Here we describe 5 new pathogenic *AMBN* variants in 11 individuals with AI. These fall within 3 groups by phenotype. Group 1, consisting of 6 families biallelic for combinations of 4 different variants, have yellow hypoplastic AI with poor-quality enamel, consistent with previous reports. Group 2, with 2 families, appears monoallelic for a variant shared with group 1 and has hypomaturation AI of near-normal enamel volume with pitting. Group 3 includes 3 families, all monoallelic for a fifth variant, which are affected by white hypoplastic AI with a thin intact enamel layer. Three variants, c.209C>G; p.(Ser70*) (groups 1 and 2), c.295T>C; p.(Tyr99His) (group 1), and c.76G>A; p.(Ala26Thr) (group 3) were identified in multiple families. Long-read *AMBN* locus sequencing revealed these variants are on the same conserved haplotype, implying they originate from a common ancestor. Data presented therefore provide further support for possible dominant as well as recessive inheritance for *AMBN*-related AI and for multiple contrasting phenotypes. In conclusion, our findings suggest pathogenic *AMBN* variants have a more complex impact on human AI than previously reported.

## Introduction

Enamel is formed when ameloblasts secrete, then mineralize, an extracellular matrix (ECM) composed of enamel matrix proteins (EMPs), in a process known as amelogenesis. Throughout the secretory stage of amelogenesis, ameloblasts secrete the EMPs amelogenin (AMELX), ameloblastin (AMBN), enamelin (ENAM), and amelotin (AMTN) and the matrix modifier matrix metallopeptidase 20 (MMP20) (Lee et al. 1996). In the later maturation stage, a further matrix modifier, kallikrein-related peptidase 4 (KLK4), is also secreted (Smith et al. 2017; Pandya and Diekwisch 2021). EMPs play an essential role in the biomineralization and structural organization of enamel (Bartlett et al. 2006).

Amelogenesis imperfecta (AI) describes a heterogeneous group of Mendelian disorders causing abnormal amelogenesis, affecting all teeth of both dentitions (Smith et al. 2017). Reported prevalence ranges between 1 in 700 (Sweden) and 1 in 14,000 (United States) (Bäckman and Holm 1986; Witkop 1988). Poor aesthetics and early functional failure create considerable challenges for affected individuals and those providing care. AI can be isolated or part of syndromic conditions, with many genes implicated (Smith et al. 2017; Wright 2023).

AMBN, a phosphorylated glycoprotein, is the second most abundant EMP after AMELX. The *AMBN* gene encodes a 447–amino acid protein that is acidic and proline rich (15.2%), a characteristic shared with other EMPs (Krebsbach et al. 1996; Pandya and Diekwisch 2021). Proline residues in EMPs are understood to inhibit formation of secondary structures such as α-helices and β-sheets. This makes EMPs intrinsically disordered proteins that do not form stable 3-dimensional (3D) structures but instead exist in heterogeneous oligomeric states (Wald et al. 2013; Vetyskova et al. 2020). This characteristic is important for amelogenesis (Stakkestad et al. 2017). Recombinant human AMBN fails to oligomerize when exon 5 is deleted (Wald et al. 2013) and mice homozygous for a deletion of *Ambn* exons 5 and 6, expressing truncated AMBN protein, produce very thin enamel (Fukumoto et al. 2005).

*AMBN* is therefore a strong candidate gene for involvement in human AI. In 2014, we reported a family with recessive hypoplastic AI due to an in-frame homozygous biallelic deletion of *AMBN* exon 6 (Poulter et al. 2014). Two further recessive AI families with biallelic *AMBN* pathogenic variants have since been reported (Prasad et al. 2016; Liang et al. 2019). A report of a large dominant family where AI and dentinogenesis imperfecta (DI) segregated with a heterozygous AMBN missense variant p.(Pro357Ser) challenged our understanding of *AMBN*-associated disease (Lu et al. 2018). Liang and colleagues suggested that, given the mixed phenotype, there could also be a variant in *DSPP*, a gene linked to *AMBN* on chromosome 4, which contains a region poorly covered by whole-exome sequencing (Liang et al. 2019). No further dominant pathogenic *AMBN* variants have been reported. This raises the possibility that the consequences of *AMBN* variants in human AI remain incompletely characterized.

Here, we report 5 novel *AMBN* variants in 11 individuals with AI that can be divided into 3 clinical groups. One has a dominant family history spanning 4 generations, and the likely causative variant in this family was also identified as monoallelic/heterozygous in 2 other apparently unrelated individuals with isolated AI. These data provide further evidence suggesting *AMBN* variants can cause both dominant and recessive AI with variations in clinical phenotypes.

## Materials and Methods

Patients were recruited though UK dental clinics in accordance with the principles of the Declaration of Helsinki (ethical approval REC 13/YH/0028). Genomic DNA was isolated from saliva or from peripheral blood by standard approaches as detailed in the Appendix methods.

Proband genomic DNA was analyzed by short-read next generation sequencing of either whole-exome sequencing (WES) or single-molecule molecular inversion probes (smMIPs) data generated on HiSeq 3000, NextSeq 500, or NextSeq 2000 sequencers (Illumina). Further details of methods used in library preparation and sequence analysis are given in the Appendix methods. The pathogenicity status of detected variants was classified according to American College of Medical Genetics and Genomics (ACMG) guidelines using Franklin (https://franklin.genoox.com) (Richards et al. 2015).

Long-read sequencing was carried out on a Flongle flowcell (R.9.4.1), using a MinION (ONT) device running MinKNOW, to analyze long-range polymerase chain reaction (PCR) products amplified by SequelPrep polymerase (ThermoFisher Scientific), following the manufacturer’s guidelines. Methods used in sequence analysis are detailed in the Appendix methods. Haplotypes were defined after selection of reference and non-reference-matching nucleotides at the positions being examined, using the Jvarkit tool biostar214299 (http://lindenb.github.io/jvarkit/Biostar214299.html) (Lindenbaum 2015). Aligned sequence reads were visualized using the Integrated Genome Viewer (v.2.7.2) (Robinson et al. 2011).

Variants were confirmed and segregation tested by PCR amplification and Sanger sequencing on an ABI3130xl Genetic Analyser (Applied Biosystems). Electropherograms were analyzed using SeqScapeTM (v.2.5) (ThermoFisher Scientific).

Intact teeth were analyzed using a high-resolution micro–computed tomography (µCT) SkyScan 1172 (Bruker) scanner to quantify mineral density. Video showing the 3D internal and external features was created using CTVox (Bruker). Longitudinal mid-bucco slices of the teeth were imaged on an S-3400N scanning electron microscope (SEM) (Hitachi). Further details are in the Appendix methods.

## Results

**Figure 1.**
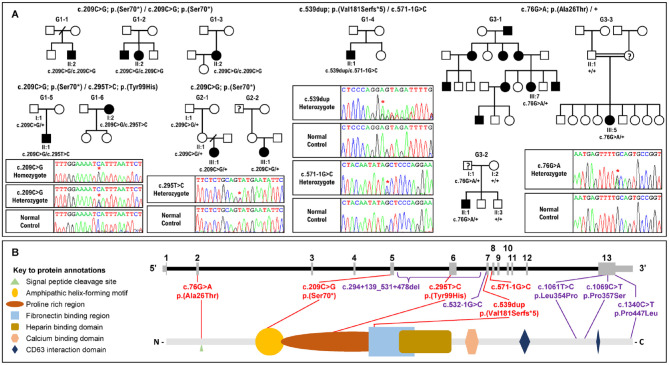
Pedigrees of the families recruited in this study, electropherograms of the variants identified and the location of the variants in the gene and protein. (**A**) Pedigrees of the 11 families described in this study with exemplar electropherograms of the variant(s) identified by whole-exome sequencing (WES) and single-molecule molecular inversion probes (smMIPs) analysis and then verified by Sanger sequencing. G1-1 and G1-3 present as cases of isolated amelogenesis imperfecta (AI) with no family history, while G1-2 consists of an affected sibling pair. Probands in these families are homozygous for the novel variant c.209C>G; p.(Ser70*) in exon 5. Families G1-5 and G1-6 also present as isolated cases, and affected individuals in each are compound heterozygotes for variants c.209C>G; p.(Ser70*) and c.295T>C; p.(Tyr99His) in exons 5 and 6, respectively. Probands from G2-1 and G2-2 present isolated AI with no apparent family history, and each carries the same heterozygous variant, c.209C>G; p.(Ser70*). G1-4 proband represents an isolated AI who is a compound heterozygote for the variants c.539dup; p.(Val181Serfs*5) and c.571-1G>C in exons 7 and 8, respectively. Three families, G3-1, G3-2, and G3-3, all carry the same heterozygous variant c.76G>A; p.(Ala26Thr) in exon 2. G3-1 proband is a single individual from a family with a clear history of dominant AI, while G3-2 and G3-3 were recruited as isolated patients with AI. G3-2, for which the father of the proband self-reported as unaffected on recruitment but was not clinically examined, carries the variant and the proband of G3-3 was reported as probable dominant AI, for which the mother was self-reporting possibly having AI. The “?” in the pedigree denotes possible AI in individuals not clinically assessed. Red stars (*) highlight nonreference nucleotides. Variant nomenclature is according to the transcript NM_016519.6. (**B**) Schematic representation of the *AMBN* gene (NM_016519.6) and its translated product showing the known domains of the 447–amino acid protein. Variants identified in this study are annotated in red; previously published variants are annotated in purple.

Probands in a large cohort of apparently unrelated patients/families with AI were subject to ongoing screening of AI-associated genes, either by targeted smMIPs or WES. Members of 11 families with likely pathogenic *AMBN* variants were identified to date, as shown in Figure 1A. All affected individuals were diagnosed with AI by experienced dental practitioners. No evidence was found of dentine changes.

### Genetic Findings

The 11 families could be sorted into 3 groups according to their *AMBN* genotypes (Table). Group 1 includes 6 families, G1-1, G1-2, G1-3, G1-4, G1-5, and G1-6, all with an *AMBN* genotype and family history consistent with autosomal recessive AI. Affected individuals from G1-1, G1-2, and G1-3 are homozygous for pathogenic variant c.209C>G; p.(Ser70*), a stop-gain mutation that is likely to undergo nonsense-mediated decay (NMD) (Kurosaki and Maquat 2016). The affected individual from a fourth family, G1-4, is compound heterozygous for a single base duplication c.539dup; p.(Val181Serfs*5) and a splice site variant, c.571-1G>C; p.(?). The c.539dup variant gives rise to a frameshift variant (in exon 7 of 13) that is predicted to undergo NMD. The variant c.571-1G>C, with a splice-AI acceptor loss score of 0.83, donor loss of 0.20, and acceptor gain of 0.79, alters the splice acceptor site for *AMBN* exon 8 and is predicted to result in exon skipping. Probands from the remaining 2 group 1 families, G1-5 and G1-6, are compound heterozygotes for the stop-gain *AMBN* variant c.209C>G; p.(Ser70*) and a novel *AMBN* missense variant c.295T>C; p.(Tyr99His). The variant c.295T>C; p.(Tyr99His) has a combined annotation dependent depletion (CADD) score of 25.8 and is classified as likely pathogenic.

**Table.** Details of AMBN Variants Detected in the Probands of 11 Recruited Families.

Group	Family ID	Family History	Phen	Method	Variant(s)						
					Transcript Change	Amino Acid Change	Zygosity	CADD	gnomAD Frequency	ACMG	ClinVar
1	G1-1	IC	HP	smMIP	c.209C>G	p.(Ser70*)	Hom	36	0.0001	Path	VCV001702585.3
	G1-2	SP	HP	WES	c.209C>G	p.(Ser70*)	Hom	36	0.0001	Path	VCV001702585.3
	G1-3	IC	HP	WES	c.209C>G	p.(Ser70*)	Hom	36	0.0001	Path	VCV001702585.3
	G1-4	IC	HP	WES	c.539dup	p.(Val181Serfs*5)	Het	22.8	Absent	Path	
					c.571-1G>C	p.?	Het	22.6	0.0001	Likely Path	VCV002444856.1
	G1-5	IC	HP	smMIP	c.209C>G	p.(Ser70*)	Het	36	0.0001	Path	VCV001702585.3
					c.295T>C	p.(Tyr99His)	Het	25.8	0.0001	Likely Path	VCV002233469.1
	G1-6	IC	HP	WES	c.209C>G	p.(Ser70*)	Het	36	0.0001	Path	VCV001702585.3
					c.295T>C	p.(Tyr99His)	Het	25.8	0.0001	Likely Path	
2	G2-1	IC	HM	smMIP	c.209C>G	p.(Ser70*)	Het	36	0.0001	Path	VCV001702585.3
	G2-2	IC	HM	smMIP	c.209C>G	p.(Ser70*)	Het	36	0.0001	Path	VCV001702585.3
3	G3-1	IC	HP	WES	c.76G>A	p.(Ala26Thr)	Het	26	Absent	Likely Path	
	G3-2	AD	HP	smMIP	c.76G>A	p.(Ala26Thr)	Het	26	Absent	Likely Path	
	G3-3	IC	HP	WES	c.76G>A	p.(Ala26Thr)	Het	26	Absent	Likely Path	

Variants are reported according to AMBN transcript NM_016519.6 and protein NP_057603.1, using human reference genome GRCh37/hg19. ACMG criteria for p.(Ser70*) and p.(Val181Serfs*5) are Path: pathogenic (PP4, PVS1, PM2), for c.571-1G>C is likely Path: likely pathogenic (PP4, PM3, PM2, PVS1), for p.(Tyr99His) is likely pathogenic (PP4, PM3, PM2, PP3), for p.(Ala26Thr) is likely pathogenic (PP4, PS4, PM2). Scoring criteria: PP3, pathogenic supporting; PP4, pathogenic supporting; PS4, pathogenic strong; PVS1, pathogenic very strong; PM2, pathogenic moderate; PM3, pathogenic moderate.

ACMG, American College of Medical Genetics; AD, autosomal dominant; CADD, combined annotation dependent depletion; ClinVar, public archive of interpretations of clinically relevant variants; gnomAD, genome aggregation database (Karczewski et al. 2020); Het, heterozygous; HM, hypomaturation; Hom, homozygous; HP, hypoplastic; IC, isolated case; Phen, phenotype; smMIP, single-molecule molecular inversion probe; SP, sibling pair; WES, whole-exome sequencing.

Group 2 includes 2 cases of isolated AI from families G2-1 and G2-2, without any history of AI in the family. The probands in each family are heterozygous for the *AMBN* variant c.209C>G; p.(Ser70*). No second *AMBN* variant was identified in *trans* in G2-1; however, the normal allele in the proband from G2-2 is a complex allele carrying 2 common *AMBN* coding variants: an in-frame deletion c.539_541del: p.(Gly180del) and the missense variant c.764C>T: p.(Ala255Val). The population allele frequencies of these variants are 0.082 and 0.086, respectively, and both are predicted to be benign. Investigation of other genes known to cause AI did not identify any potentially relevant pathogenic variants.

Group 3 includes family G3-1, with an extensive family history of dominant AI but only a single affected individual recruited. This group also includes 2 additional families with possible AD-AI: G3-2, for which the father of the proband self-reported as unaffected but was not clinically examined, and G3-3, for which the proband’s mother is said to have AI but was not examined clinically. Probands from these families are all heterozygous carriers of the novel missense variant c.76G>A; p.(Ala26Thr). This variant was classified as likely pathogenic. No second *AMBN* variant was identified on the normal allele in these families.

### Founder Effect Screening

The presence of variant c.209C>G; p.(Ser70*) in 7 families (groups 1 and 2), c.295T>C; p.(Tyr99His) in 2 families (group 1), and c.76G>A; p.(Ala26Thr) in 3 families (group 3) suggests these variants may have been inherited from a common ancestor. To test this hypothesis, we examined the haplotype backgrounds of these variants at the *AMBN* locus, using long-range PCR and third-generation nanopore sequencing.

**Figure 2.**
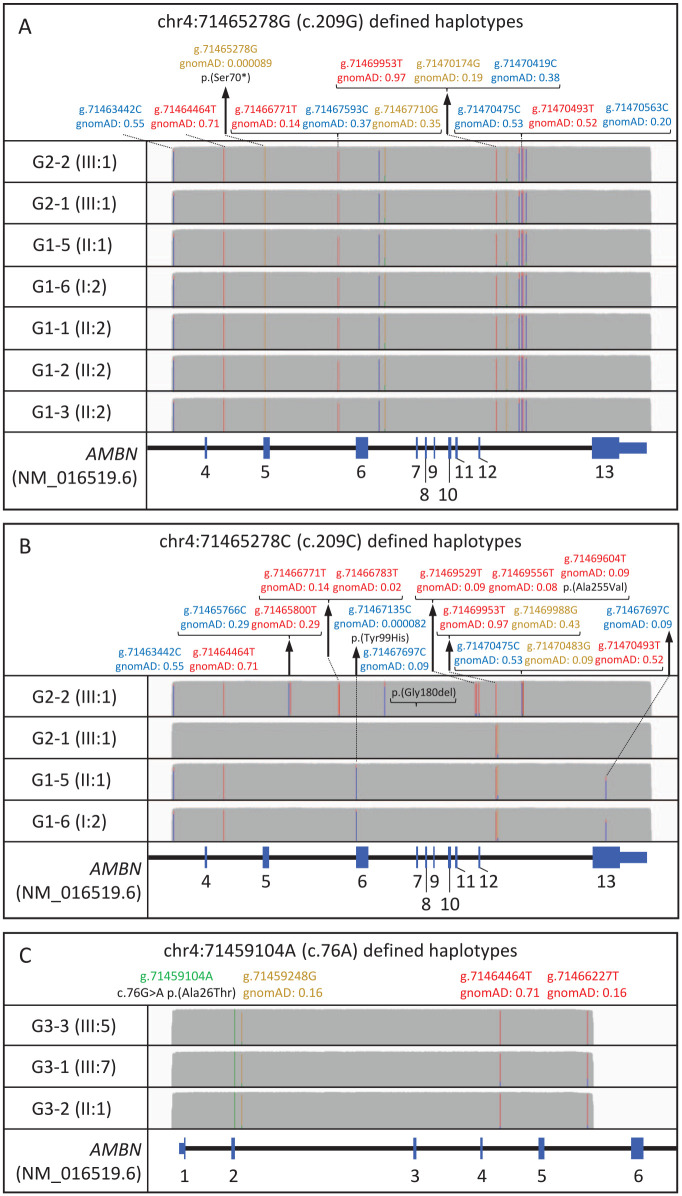
Long-read sequencing of a 9.7-kb amplification product from the *AMBN* locus spanning exons 4 to 12 of *AMBN*. Sequence analysis reveals the founder haplotype backgrounds on which variants c.209C>G; p.(Ser70*) and c.295T>C; p.(Tyr99His) have arisen, which are shared by all the families that carry them. Nucleotide positions are reported according to human genome build hg19. Allele frequencies are from the gnomAD database v.3.1.2 and are based on high-quality genotypes from a data set of 76,156 samples. The IGV allele frequency threshold is 0.6. Figure created using IGV version 2.12.2, with y-axis coverage tracks scaled to 2,300×. (**A**) Ten alleles identified from 3 homozygous (G1-1, G1-2, and G1-3), 2 heterozygous (G2-1 and G2-2), and 2 compound heterozygous (G1-5 and G1-6) individuals, all bearing the c.209C>G; p.(Ser70*) variant on a shared 11 nonreference single-nucleotide polymorphism (SNP) haplotype background. (**B**) The alleles not carrying the c.209C>G; p.(Ser70*) variant (wild type alleles) in families G2-1, G2-2, G1-5, and G1-6 tagged by the reference C nucleotide at position c.209 have haplotype backgrounds distinct from those bearing the pathogenic variants. Shared haplotype for the 2 alleles bearing the pathogenic variant c.295T>C; p.(Tyr99His) in families G1-5 and G1-6, each on the same background haplotype of 5 nonreference SNPs. (**C**) Haplotype background of the variant c.76G>A; p.(Ala26Thr) in families G3-1, G3-2, and G3-3 is identical, consisting of 3 other nonreference variants.

A 9,681-bp DNA segment spanning exons 4 to 13 of *AMBN* and including amino acid residues 70 (exon 5) and 99 (exon 6) was PCR amplified and analyzed by long-read sequencing in probands from families G1-1, G1-2, and G1-3 (homozygous for c.209C>G; p.(Ser70*)), G1-5 and G1-6 (compound heterozygotes for c.209C>G; p.(Ser70*) and c.295T>C; p.(Tyr99His)), and G2-1 and G2-2 (heterozygotes for c.209C>G; p.(Ser70*)). We observed a haplotype consisting of 11 nonreference nucleotides arranged in *cis* with c.209C>G; p.(Ser70*) in all 7 samples (Fig. 2A). The c.295T>C; p.(Tyr99His) variant was found on a different haplotype background characterized by 5 nonreference nucleotides in the 2 families carrying it (Fig. 2B).

An 8,520-bp amplicon, spanning exons 1 to 5 and including residue 26, was PCR amplified and analyzed by long-read nanopore sequencing in probands from families G3-1, G3-2, and G3-3, all heterozygous for the *AMBN* variant c.76G>A; p.(Ala26Thr). These data revealed a haplotype shared by all 3 families that comprised 3 nonreference nucleotides (Fig. 2C).

### Phenotyping

**Figure 3.**
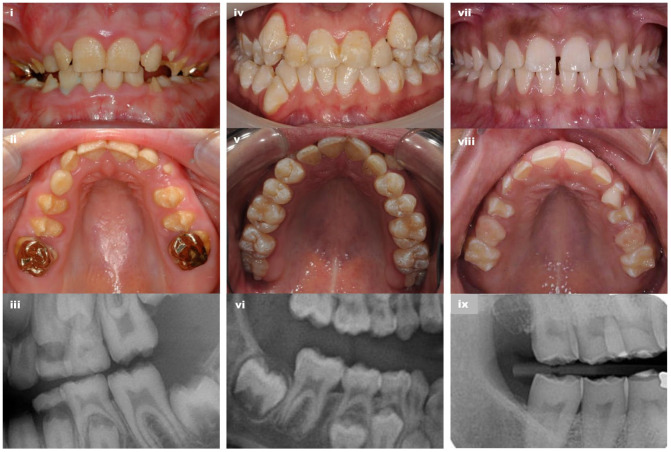
Clinical images and radiographs of the teeth capture the differences between the 3 groups. Group 1 (i–iii): Yellow hypoplastic amelogenesis imperfecta (AI) reflects the absence of any meaningful enamel on dental radiography (i and ii G1-2; iii G1-5 bitewing). Group 2 (iv–vi): Hypomaturation AI is characterized by variations in color with pits and other localized morphological changes that disrupt the normal clinical enamel surface. Dental radiography confirms near-normal enamel volumes with a clear difference between enamel and dentine radiodensity (iv and v G2-2; vi G2-2 detail from panoramic radiograph). Group 3 (vii–ix): White hypoplastic AI reflects the presence of a thin layer of enamel on dental radiography (vii and viii G3-3; ix G3-1 detail from panoramic radiograph). Further clinical images are included in Appendix Figure 1.

Images of teeth and dental radiographs identified differences in the clinical phenotypes of the 3 groups recognized through genetic analyses (Fig. 3 and Appendix Fig. 1). Affected individuals in group 1 were characterized by hypoplastic AI with poor-quality enamel, with teeth having a yellow appearance following early posteruption loss of a thin layer of creamy, opaque mineralized tissue. Affected individuals in group 3 also had hypoplastic AI, but this differed from group 1 through the presence of a thin layer of more persistent enamel, which gives the teeth a whiter long-term appearance than group 1. Group 2 has a very different phenotype, characterized by hypomaturation AI with associated pits and minor morphological variations within a near-normal enamel volume that is more radio-dense than the supporting dentine on clinical radiography. No clear dentine abnormalities were evident on dental radiographs.

**Figure 4.**
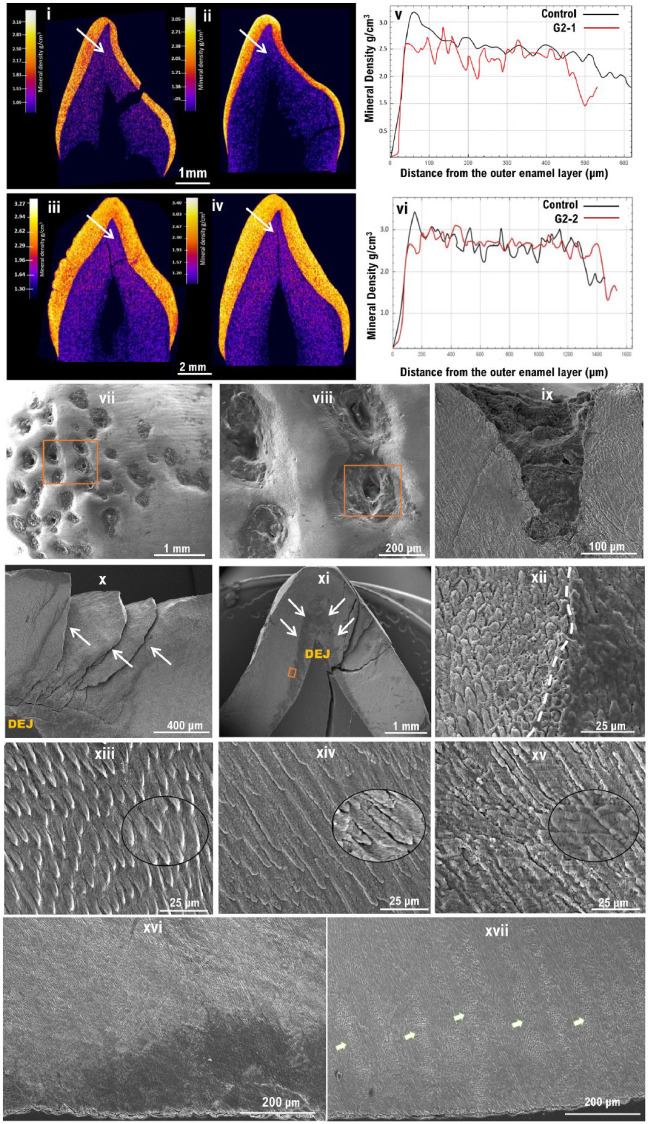
Phenotypic characterization of the teeth of group 2 families. (i–iv) Micro–computed tomography (µCT): False-colored calibrated heatmaps. (i) A primary upper lateral incisor from the affected individual from G2-1 and (iii) a permanent canine from G2-2. Teeth of the same types obtained from unrelated unaffected individuals were used as controls for comparison and are shown in (ii) and (iv), respectively. (i, iv) No significant differences in enamel volume was observed between affected and healthy controls, but the mineral density distribution was found to be disturbed. (v, vi) Mineral density line scans showing the distribution of enamel density form the enamel surface to the dentine–enamel junction (DEJ) in G2-1 and G2-2, respectively, as shown by the arrows in i–iv. In both controls, note the initial high peaks present at the surface and the gradual decrease of mineral density toward the DEJ, which are absent in the amelogenesis imperfecta–affected teeth confirming the disturbed mineral density observed in the µCT images. (iii) G2-2 tooth shows an uneven, pitted surface that is absent from the control tooth. (vii–xvii) Scanning electron microscope images: (vii–viii) Surface topography features in G2-2 showing numerous clear pitting of the labial enamel surface, extending through the enamel (ix) and to the DEJ (x). (xi) Labiolingual section of G2-2, a more disrupted inner enamel below the cuspal area is shown by the white arrows. (xii) Higher magnification of the inset in (xi) showing a clear demarcation (white dotted line) between a less dense enamel located near the DEJ and the rest of the enamel. (xiii–xv) Higher magnification of the enamel microstructure at ×1k, with ×3.5k insets to illustrate prismatic structure as well as crystallite orientation. (xiii) Healthy enamel. (xiv–xv) Affected teeth from the family G2-1 and G2-2, respectively. (xiv–xv) Note the poorly formed enamel rods that appear fused at many areas, making it difficult to distinguish the boundaries between rod and interrod regions. (xvi) Low magnification of G2-2 showing missing Hunter–Schreger banding as opposed to the healthy enamel (arrows) in (xvii).

Teeth were available from a primary upper lateral incisor from the G2-1 proband and a permanent canine from the G2-2 proband for laboratory analyses. µCT of these teeth revealed normal enamel volume (Fig. 4i –iv). No significant differences were observed in average enamel mineral density (EMD) between affected and control teeth of the same type obtained from unrelated unaffected individuals. The EMD in G2-1 and its respective control were 2.561 g.cm^–3^ and 2.546 g.cm^–3^, and in G2-2 and its respective control, they were 2.569 g.cm^–3^ and 2.721 g.cm^–3^. An outer layer of particularly high mineral density seen in the control was missing in the G2-1, while the enamel of the G2-2 appeared pitted (Fig. 4i, iii) with pits extending through the enamel layer to the dentine–enamel junction (DEJ) (Fig. 4vii –ix, Appendix video). SEM analysis of these teeth showed disrupted, poorly formed prismatic microstructure with little demarcation between rod and interrod regions (Fig. 4xiii–xv). Hunter–Schreger banding was also absent in the affected tooth (Fig. 4xvi) as opposed to the control (Fig. 4xvii).

## Discussion

The data presented support *AMBN* variants having a complex impact on human AI that highlights our incomplete under-standing.

Six of the families described here have genotypes and inheritance patterns consistent with autosomal recessive hypoplastic AI with poor-quality enamel. Clinical images were consistent with rapid failure of a thin creamy mineralized tissue after eruption, leaving a predominantly yellow appearance. These group 1 families fit with previous reports of recessive AI due to biallelic pathogenic variants in *AMBN*, with no clinically significant enamel changes in heterozygous carriers (Poulter et al. 2014). Affected individuals from 5 of the 6 group 1 families were homozygous (*n* = 3) or heterozygous (*n* = 2) for the c.209C>G p.(Ser70*) variant, which occurred on the same haplotype consistent with a common UK founder allele. This variant is predicted to produce no AMBN protein. In the 2 families in whom heterozygous c.209C>G p.(Ser70*) was paired with the missense c.295T>C p.(Tyr99His) change, this also resulted in a poor enamel quality form of hypoplastic AI.

Intriguingly, group 2 included isolated AI cases heterozygous for *AMBN* c.209C>G; p.(Ser70*), with a very different AI phenotype from group 1 families. Heterozygosity for p.(Ser70*) alone being sufficient to cause AI is inconsistent with the existence of many apparently unaffected heterozygous carriers in group 1 families. Individuals G1-5 I-1 and G2-1 II-1, who are heterozygous carriers of p.(Ser70*), consented to give DNA but were not subject to clinical examination for possible subtle enamel developmental abnormalities, although neither was flagged as having AI. In contrast, probands in group 2 families were diagnosed clinically as having AI. Full gene screening of *AMBN* by long-read sequencing did not detect any second pathogenic variants in the coding sequence, introns, or 5′ or 3′ UTRs, and there was no evidence of allele dropout, which might imply the presence of structural variants missed by short-read sequencing in either of these 2 families (Appendix Fig. 2). The *trans* allele in G2-2 was found to carry 2 further coding variants that could act as hypomorphic alleles contributing to AI in G2-2 but are too common to be pathogenic themselves, even when homozygous. It is therefore necessary to consider alternative hypotheses, either that there may be other hypomorphic variants on the second *AMBN* alleles in G2-1 and G2-2, which have not been detected in genetic screening, or that genetic or environmental modifiers combine with the p.(Ser70*) variant to cause disease. Clinical evaluation of individuals presenting with whole-dentition abnormal enamel development considered possible environmental factors such as dental fluorosis or major systemic illness, before a clinical diagnosis of AI was made (Wright 2023). This does not preclude some attenuation by environmental factors of what is primarily a genetically driven enamel phenotype. No potentially pathogenic variants were identified in other known AI genes, but noncoding variants in known genes or variants in previously undiscovered amelogenesis-related genes could be present. However, assessing the relative contributions of genes and environment to severity in AI would require a large patient cohort and is beyond the scope of this study.

Group 3 includes 3 families with the same heterozygous variant, c.76G>A; p.(Ala26Thr), which is absent from gnomAD. Of these, G3-1 has a clear family history of dominantly inherited AI. However, only a single family member was recruited, so it was not possible to confirm cosegregation of the variant with AI. Observation of 2 other apparently unrelated patients with AI (G3-2 and G3-3) with the same variant on the same founder haplotype implies descent from a common ancestor, which is further evidence of cosegregation of this variant with AI as a dominantly inherited trait. No potentially pathogenic second *AMBN* variant was identified in smMIPs, WES, or long-read nanopore sequencing data in these individuals.

The spectrum of pathogenic variants in *AMBN* revealed by this and previous studies encompasses a premature termination codon (PTC), a 1-bp deletion leading to a frameshift, 2 splice acceptor site variants, an in-frame whole-exon deletion, and 5 missense variants. The consequences of these variants have not been determined experimentally, but it seems likely that the PTC, frameshift, splice variants, and exon deletion will act as null alleles, reducing the amount of functional AMBN available during amelogenesis. Teepe and coworkers reported that transgenic mice expressing Ambn at concentrations lower or higher than the wild-type level had enamel abnormalities (Teepe et al. 2014), suggesting that a specific AMBN concentration is crucial for amelogenesis. Furthermore, group 1 families G1-1, G1-2, and G1-3 are homozygous for likely null variants, providing additional support for the interpretation that lack of intact AMBN is the likely disease mechanism in these cases.

For missense variants, the disease mechanism is less clear, but they may also be functional knockouts. The p.(Tyr99His) substitution changes an aromatic tyrosine to a basic histidine in the first of 15 highly conserved amino acid residues in the proline-rich region of AMBN. This region is retained in AMBN isoform I (ISOI) but removed in isoform II (ISOII) due to alternative splicing, and both isoforms are highly conserved and are coexpressed in vitro, suggesting they might perform different functions during enamel development (MacDougall et al. 2000; Vetyskova et al. 2020). Probands in families G1-5 and G1-6, who are compound heterozygotes for p.(Ser70*) and p.(Tyr99His), had thin poor-quality enamel, similar to families homozygous for p.(Ser70*). It is therefore likely that p.(Tyr99His) is also a functional knockout.

On clinical examination, group 1 enamel is yellow, thin, and of poor quality without normal microstructure (Poulter et al. 2014). By contrast, group 3 enamel associated with c.76G>A; p.(Ala26Thr) is thin on radiography, yet the white appearance of the teeth is consistent with little posteruption breakdown. Without laboratory analyses, it is unknown if this has a normal enamel microstructure. This substitution changes a nonpolar hydrophobic alanine (conserved in all mammals except the toothless platypus) at the C-terminal amino acid of the AMBN secretory signal peptide, immediately adjacent to the cleavage site, to a polar hydrophilic threonine (Delsuc et al. 2015). Proteins such as AMBN that are destined for the extracellular environment are transported to the endoplasmic reticulum (ER) under the direction of the signal peptide, which is cleaved from the protein before secretion. Many human diseases, including AI, are caused by ER stress, resulting from the misfolding of newly synthesized proteins as they are trafficked through the ER (Brookes et al. 2017; Morikawa and Urano 2022). It is therefore plausible that the apparently dominant hypoplastic AI phenotype associated with the p.(Ala26Thr) signal peptide variant arises by a different disease mechanism compared to null variants causing recessive AI. A dominant negative effect may lead to impairment of the normal ameloblast secretory pathway, ER stress, and ultimately ameloblast apoptosis.

Accordingly, these data contribute to the debate as to whether mutations in *AMBN* cause dominant as well as recessive AI. A large dominant family with a combined AI/DI phenotype was segregated with the heterozygous *AMBN* missense variant p.(Pro357Ser) (Lu et al. 2018). This report was questioned by Liang and coworkers (2019), who cautioned that the diagnosis may be DI caused by a variation in *DSPP* (a gene linked to *AMBN* on chromosome 4 and with a repetitive region that is difficult to sequence by WES). However, a crossover below marker D4S2931, visible in individual IV7 in the microsatellite data presented but not discussed by Lu and colleagues (2018), clearly excludes the possibility that a variant in the *DSPP* gene, 17 Mb distal to *AMBN*, could cause the phenotype seen in this family. Furthermore, the families described in our study were diagnosed with AI in the absence of any clear dentine abnormalities, meaning that specific exclusion of *DSPP* or other genes involved in DI in these families is not required. The patterns of inheritance in these families, together with evidence that they share a common ancestor, therefore provide additional support for dominant inheritance of AI due to variants in *AMBN*.

These data provide new insight into how null *AMBN* variants contribute to human AI and emphasize the importance of AMBN concentration during amelogenesis. Data are also presented consistent with a very specific, rare heterozygous missense change causing a type of AI distinctive from that due to loss of function, consistent with dominant inheritance. It is plausible that *AMBN* variants will not only cause different types of AI but may also influence enamel formation in other situations that are of relevance to enamel failure and how it is clinically managed.

## Author Contributions

U. Hany, C.M. Watson, C.F. Inglehearn, A.J. Mighell, contributed to conception, design, data acquisition, analysis, and interpretation, drafted and critically revised the manuscript; L. Liu, G. Nikolopoulos, C.J. Brown, A. Patel, H.D. Rodd, R. Balmer, A. Harfoush, M. Al-Jawad, contributed to data acquisition and interpretation, critically revised the manuscript; C.E.L. Smith, J.A. Poulter, contributed to conception, design, data acquisition and interpretation, critically revised the manuscript. All authors gave final approval and agree to be accountable for all aspects of the work.

## Supplemental Material

sj-docx-1-jdr-10.1177_00220345231203694 – Supplemental material for Novel Ameloblastin Variants, Contrasting Amelogenesis Imperfecta Phenotypes Supplemental material, sj-docx-1-jdr-10.1177_00220345231203694 for Novel Ameloblastin Variants, Contrasting Amelogenesis Imperfecta Phenotypes by U. Hany, C.M. Watson, L. Liu, G. Nikolopoulos, C.E.L. Smith, J.A. Poulter, C.J. Brown, A. Patel, H.D. Rodd, R. Balmer, A. Harfoush, M. Al-Jawad, C.F. Inglehearn and A.J. Mighell in Journal of Dental Research

sj-pptx-1-jdr-10.1177_00220345231203694 – Supplemental material for Novel Ameloblastin Variants, Contrasting Amelogenesis Imperfecta Phenotypes Supplemental material, sj-pptx-1-jdr-10.1177_00220345231203694 for Novel Ameloblastin Variants, Contrasting Amelogenesis Imperfecta Phenotypes by U. Hany, C.M. Watson, L. Liu, G. Nikolopoulos, C.E.L. Smith, J.A. Poulter, C.J. Brown, A. Patel, H.D. Rodd, R. Balmer, A. Harfoush, M. Al-Jawad, C.F. Inglehearn and A.J. Mighell in Journal of Dental Research
