# Supplementary material for: Novel Ameloblastin Variants, Contrasting Amelogenesis Imperfecta Phenotypes
Source: J Dent Res. 2023 Dec 6;103(1):22–30. doi: 10.1177/00220345231203694 (PMC10734210; doi:10.1177/00220345231203694)
Supplement: sj-docx-1-jdr-10.1177_00220345231203694 – Supplemental material for Novel Ameloblastin Variants, Contrasting Amelogenesis Imperfecta Phenotypes [file sj-docx-1-jdr-10.1177_00220345231203694.docx]

**Novel ameloblastin variants, contrasting Amelogenesis Imperfecta phenotypes.**

**Authors**

Ummey Hany^1^, Christopher M. Watson^1,2^, Lu Liu^1,3^, George Nikolopoulos^1^, Claire E. L. Smith^1^, James A. Poulter^1^, Catriona J. Brown^4^, Anesha Patel^5^, Helen D Rodd^6^, Richard Balmer^3^, Asmaa Harfoush^3^, Maisoon Al-Jawad^3^, Chris F. Inglehearn^1,7^, Alan J. Mighell^3,7^

**Affiliations**

1: Leeds Institute of Medical Research, University of Leeds, St James's University Hospital, Leeds, LS9 7TF, UK

2: North East and Yorkshire Genomic Laboratory Hub, Central Lab, St. James's University Hospital, Leeds, LS9 7TF, UK

3: School of Dentistry, Clarendon Way, University of Leeds, Leeds, UK

4: Birmingham Dental Hospital, Mill Pool Way, Edgbaston, Birmingham, UK

5: LCRN West Midlands Core Team, NIHR Clinical Research Network (CRN), Birmingham Research Park (West Wing), Vincent Drive, Edgbaston, Birmingham, UK

6: Academic Unit of Oral Health Dentistry and Society, School of Clinical Dentistry, University of Sheffield, Sheffield, S10 2TA, UK

7: Joint senior authors

1. **Supplementary Methods**

**Patient recruitment**

Patients were recruited through UK paediatric dental clinics. Written consent was obtained in accordance with the principles of the Declaration of Helsinki and local ethical approval was granted for this study (REC 13/YH/0028). Genomic DNA was isolated from saliva samples using Oragene® sample collection tubes (DNA Genotek Inc. Ontario, Canada) and following manufacturer's instructions, or from peripheral blood lymphocytes using either a Chemagic 360 (Perkin Elmer, Waltham, MA, USA) or standard salting-out techniques.

**Whole exome sequencing**

Two wet-laboratory workflows were used to perform whole exome sequencing (WES), these comprised either the SureSelect Human All Exon v6 kit (Agilent Technologies, Wokingham, UK) or the Human Comprehensive Exome kit (10-50 Mb) (Twist Bioscience, San Francisco, CA, USA) following manufacturer’s protocols throughout. SureSelect libraries were sequenced on a HiSeq 3000 (Illumina Inc., San Diego, CA, USA) which generated paired-end 150 bp reads. For the Twist workflow, sequencing was carried out using a P3 flowcell on a NextSeq 2000 (Illumina Inc.) which also generated paired-end 150 bp reads.

Confirmation of raw read quality was performed using FastQC (v.0.11.3) (https://www.bioinformatics.babraham.ac.uk/projects/fastqc/), before sequence reads were aligned to an indexed human reference genome (build hg19) using BWA (v.0.7.12-r1.39) (https://bio-bwa.sourceforge.net/) (Li and Durbin 2009). PCR duplicates were removed using Picard (v.2.5.0) (https://broadinstitute.github.io/picard/), before non-reference bases were identified using the Genome Analysis Tool Kit (GATK) HaplotypeCaller (v.3.5) (https://gatk.broadinstitute.org) according to recommended best practice workflows (DePristo et al. 2011). Identified sequence variants were annotated with functional and population frequency data using the Variant Effect Predictor (VEP) (v.83) (McLaren et al. 2016). Allele frequencies are reported according to the Genome Aggregation Database (gnomAD; v.2.1.1) (https://gnomad.broadinstitute.org/) (Karczewski et al. 2020) and splicing predictions are according to a web-lookup implementation of Splice-AI (https://spliceailookup.broadinstitute.org) (Jaganathan et al. 2019).

**Single-molecule molecular inversion probes (smMIPs) sequencing**

smMIPs covering the coding sequences of 19 genes (Table S1) associated with non-syndromic AI were designed using MIPGEN (https://github.com/shendurelab/MIPGEN) (Boyle et al. 2014) and synthesized by Integrated DNA Technologies (IDT; Leuven, Belgium) before being pooled in equimolar ratios. The smMIPs pool was next 5'-phosphorylated and 100 ng of each genomic DNA was subjected to targeted capture and ligation using the phosphorylated probe pool that was diluted to a ratio of 800 smMIPs copies for each DNA molecule in the final capture reaction. Sequencing was carried out using a Nextseq 500 (Illumina Inc.) generating paired-end 150 bp reads.

Sample demultiplexing and the removal of unique molecular identifiers (UMIs) was performed using a local implementation of the MIPVAR pipeline (https://sourceforge.net/projects/mipvar/) which was modified for compatibility with local computing hardware. This enabled sample demultiplexing and the removal of unique molecular identifies prior to ligation- and extension-arm processing using standard tools (BWA- v.0.7.12 (human reference genome build hg19), Picard v.1.102.0 and the GATK HaplotypeCaller v.3.2-2). Variants were recorded in VCF format and annotated using VEP.

**Variant interpretation**

The pathogenicity status of detected variants was classified according to the American College of Medical Genetics and Genomics (ACMG) guidelines using Franklin (https://franklin.genoox.com) (genoox, Palo Alto, CA ,USA) (Richards et al. 2015).

**Long-read sequencing**

Primer3 (http://primer3.ut.ee/) was used to design PCR primers which were synthesized by IDT (Leuven, Belgium). Two different amplicons were sequenced to cover the full AMBN gene; first one spanning exons 1 to 5 including 100bp of the promoter region, targeted a region of 8520 bp and the second one covered exons 4 to 13 targeted a region of 9681 bp. Primers for the first amplicon were, forward /CAATGTCCCTGCACGCAATA and reverse /GCAAGGAAGTCTCGCAACAA. Primers for the second amplicon were: forward /AAGCTGGGGGCAGTCAATAC and reverse /AGCAAAGGTAGAGGATGAGTATGC. Long-range PCR was performed using the SequelPrepTM polymerase (ThermoFisher Scientific), following the manufacturer’s guidelines. Sequencing libraries were prepared using the SQK-LSK109 ligation kit (Oxford Nanopore Technologies (ONT), Oxford, UK). A 24-hr sequencing run was initiated for each sample on a Flongle flowcell (R.9.4.1) using a MinION (ONT) device running MinKNOW.

Guppy (v.5.0.16) (https://nanoporetech.com/) was used to perform basecalling, which converted raw data from fast5 to FASTQ.gz format. Sequencing adaptors were removed using Porechop (v.0.2.4) (https://github.com/rrwick/Porechop) before NanoFilt (v.2.8.0) (De Coster et al., 2018) was used to select reads by length (± 500 bp surrounding the expected size of the amplified fragment) and quality (Q≥10) (https://github.com/wdecoster/nanofilt). Processed reads were aligned to the human reference genome (build hg19) using MiniMap2 (v.2.22) (https://github.com/lh3/minimap2). Haplotypes were defined following the selection of reference and non-reference matching nucleotides at position chr4:70599561 using the Jvarkit tool biostar214299 (http://lindenb.github.io/jvarkit/Biostar214299.html) (Lindenbaum 2015). Samtools (v.1.14) was used to downsample corresponding BAM files to 2,000×. Aligned sequence reads were visualised using the Integrated Genome Viewer (v.2.7.2) (Robinson et al. 2011).

**Sanger sequencing verification**

Primer pairs were designed using AutoPrimer3 (https://github.com/gantzgraf/autoprimer3) and synthesized by IDT (Leuven, Belgium). Q5® High-Fidelity 2X Master Mix (New England Biolabs, Ipswich, MA, USA) was used for PCR amplification, which was confirmed by agarose gel electrophoresis. PCR products were purified using ExoSAP-IT (ThermoFisher Scientific, Waltham, MA, USA) prior to Sanger sequencing using BigDye Terminator v.3.1 and resolved on an ABI3130xl Genetic Analyser (Applied Biosystems, Paisley, UK). Electropherograms were analysed using SeqScapeTM (v.2.5) (ThermoFisher Scientific).

**Micro-computed tomography (µCT)**

Intact teeth were analysed using a high-resolution micro-computed tomography (µ-CT) SkyScan 1172 (Bruker, Belgium) scanner to quantify mineral density. Mineral density values were calculated relative to three hydroxyapatite standards, of 0.25 and 0.75 g/cm3 (Bruker, Belgium), and 2.9 g/cm3 (Himed, USA). Fiji/Image J was used to analyse enamel density using a pixel threshold above 2.0 g/cm3. Video showing the 3D internal and external features were created using CTVox (Bruker, Belgium).

**Scanning electron microscopy (SEM)**

Longitudinal mid-bucco slices of the teeth were obtained using an Accutom 10 cutting machine and diamond cutting wheel (Struers, Germany). After removing surface debris, slices were gold coated (Agar Scientific, Elektron Technology, UK). Imaging was performed by S-3400N (Hitachi, Japan) SEM. References

1. **Supplementary Figures**

**Figure S1:** Clinical images and dental radiographs available for Group 1 (G1-1, G1-2, G1-3, G1-4, G1-5 and G1-6), Group 2 (G2-1 and G2-2) and Group 3 (G3-1, G3-2 and G3-3) families.

**Images and radiographs from group 1 families**

Probands from group 1 families (G1-1, G1-2, G1-3, G1-4, G1-5 and G1-6) display yellow hypoplastic AI, reflecting an absence of meaningful enamel obvious in dental radiography in G1-1 and G1-5.

**
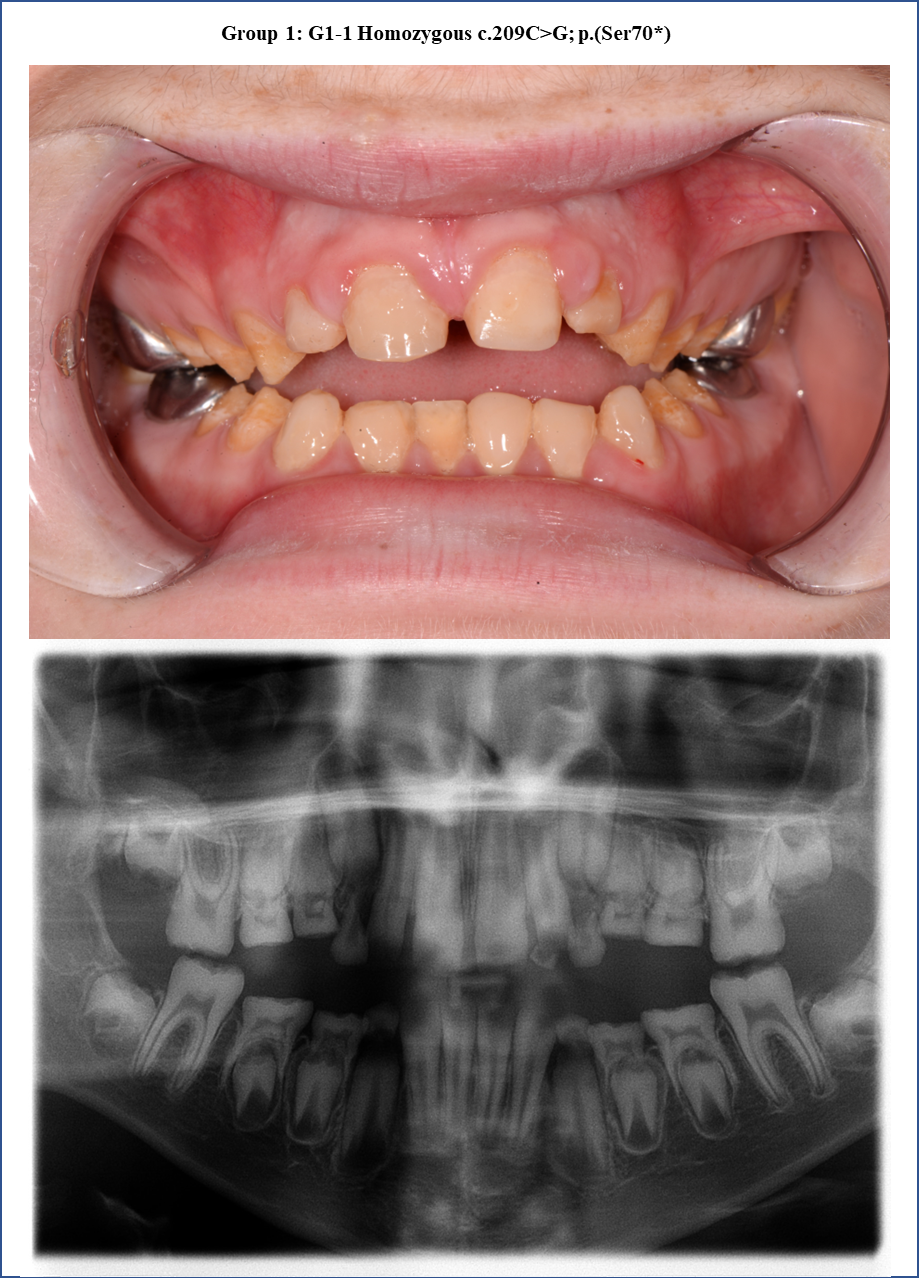
**


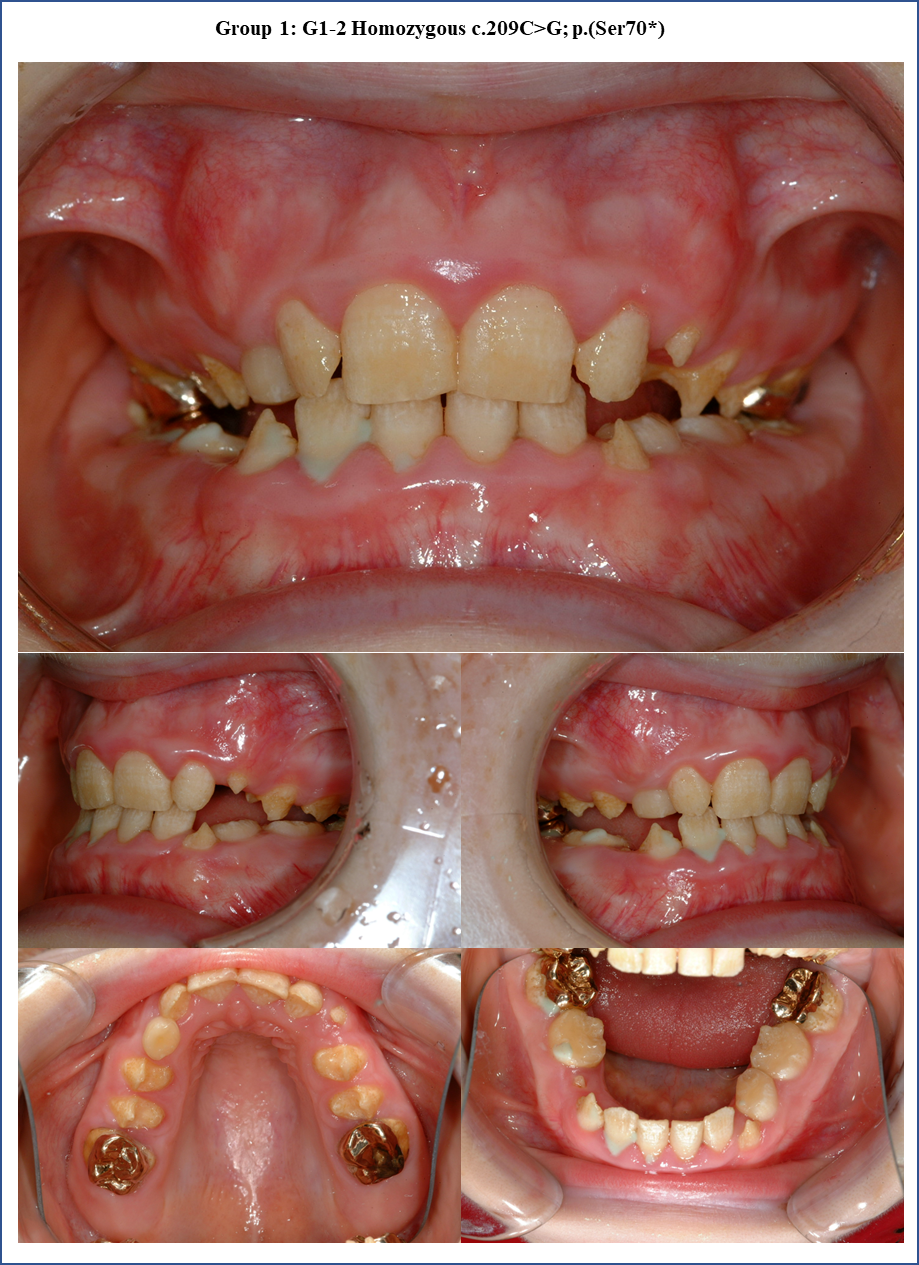


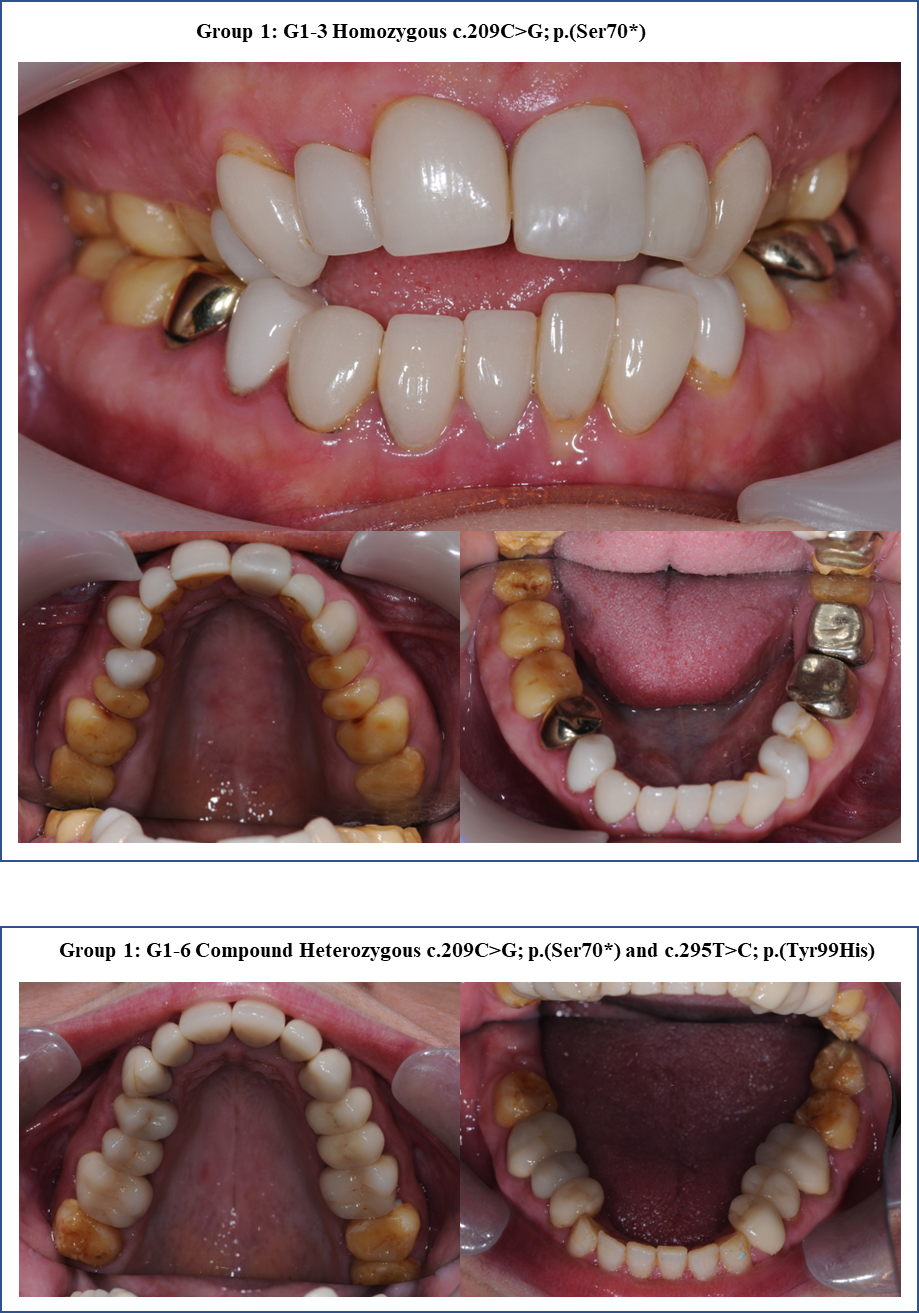


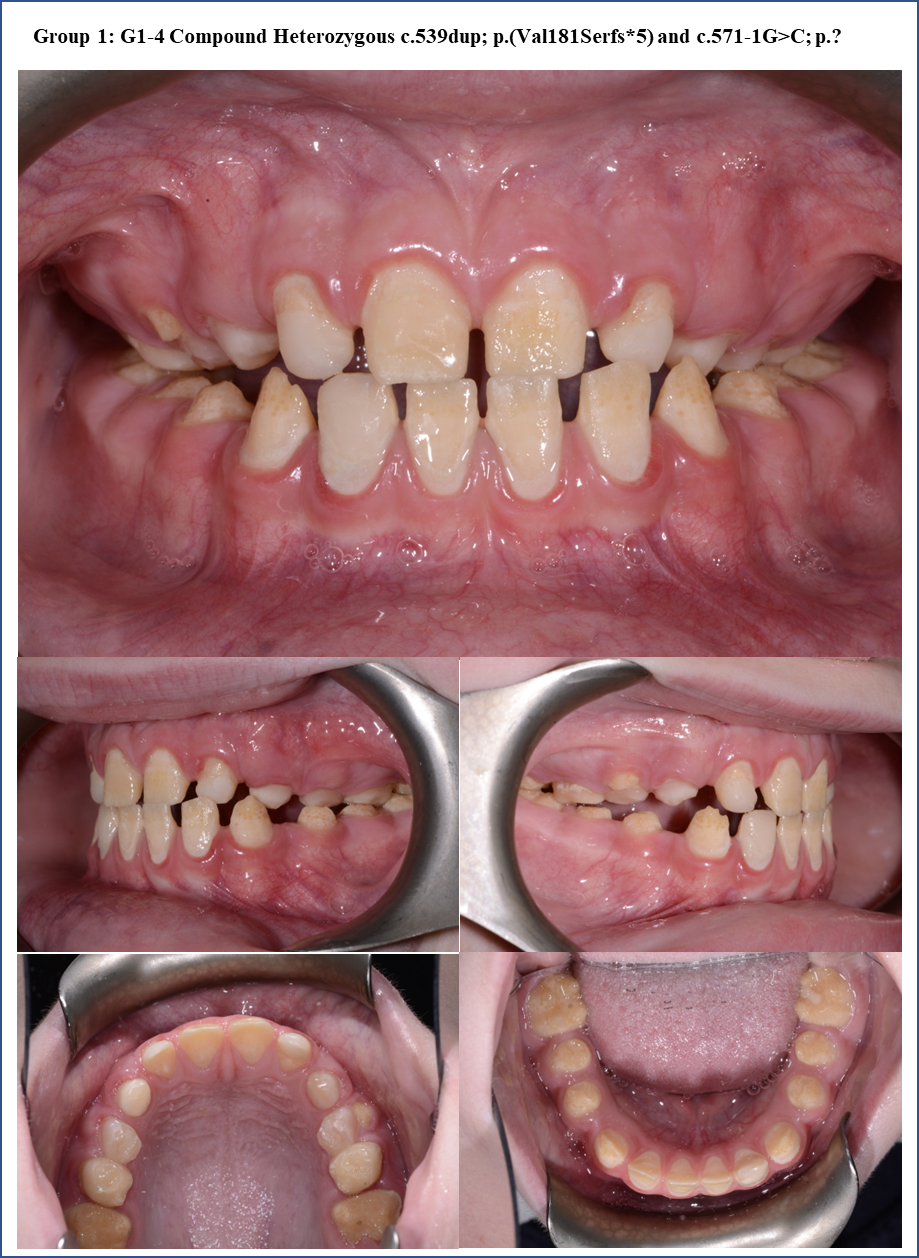


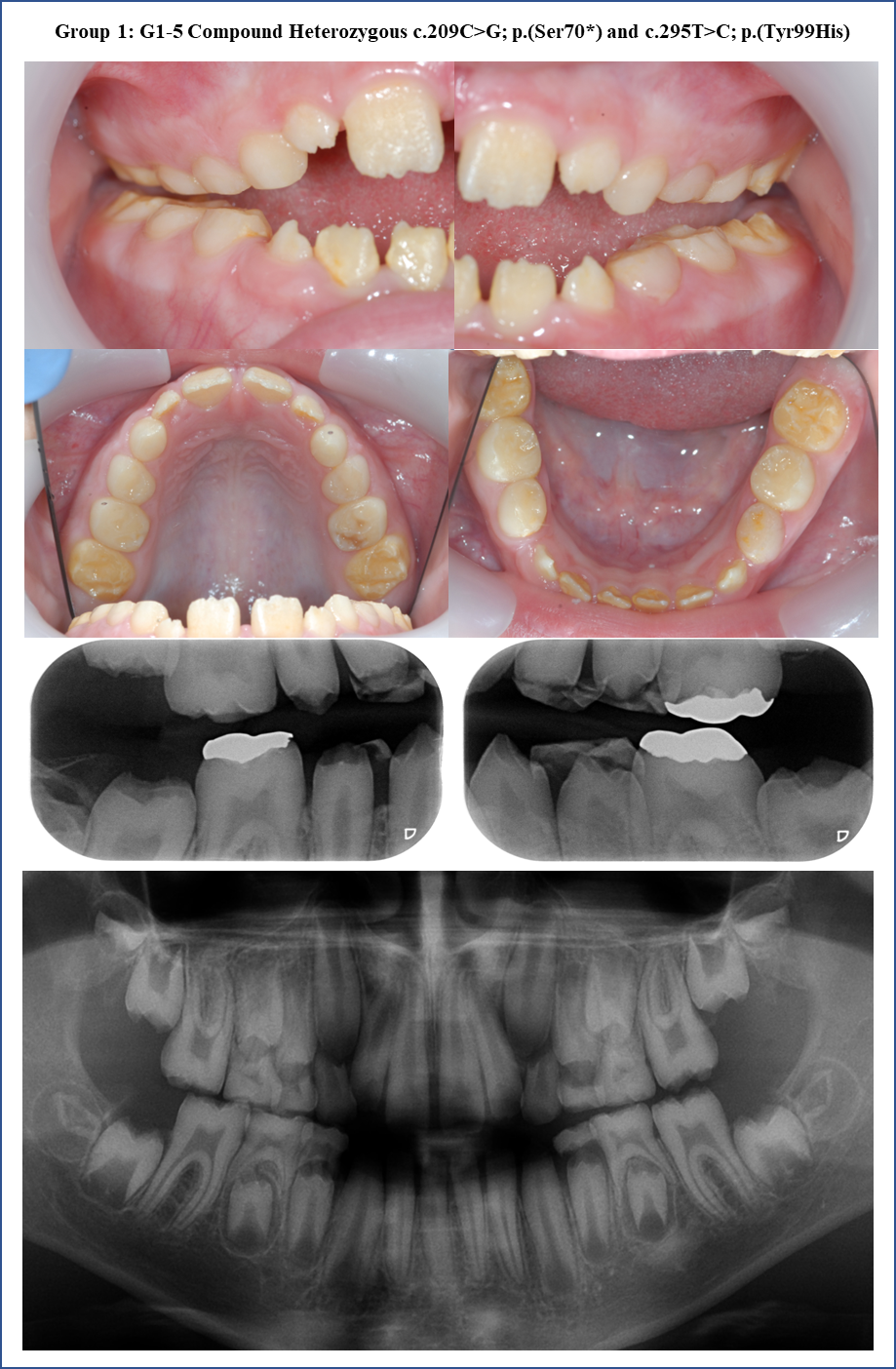


**Images and radiographs from group 2 families**

Probands from G2-1 and G2-2 demonstrate hypomaturation AI, characterised by variations in colour with pits and other localised morphological changes that disrupt the normal clinical enamel surface.


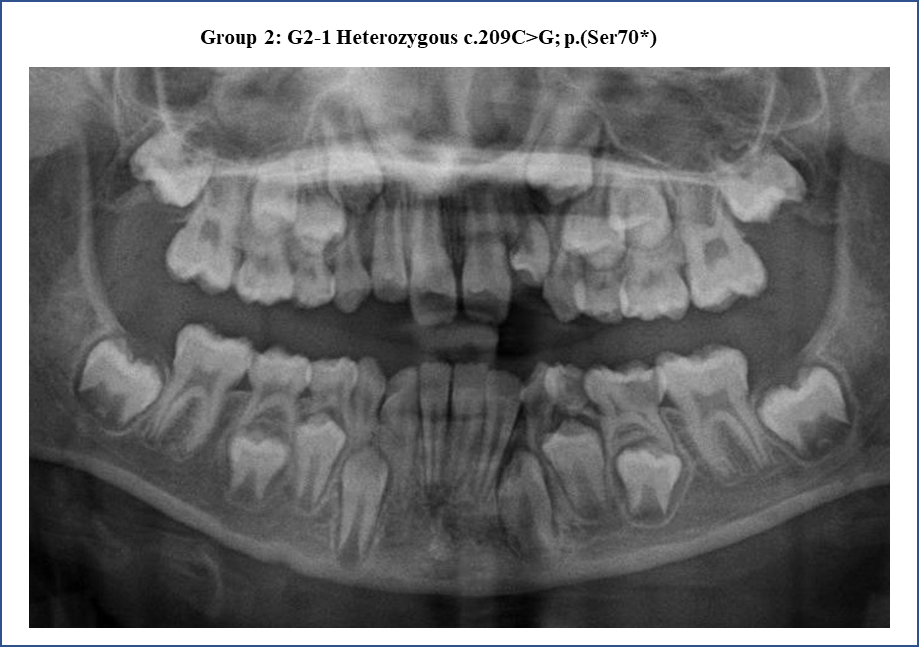


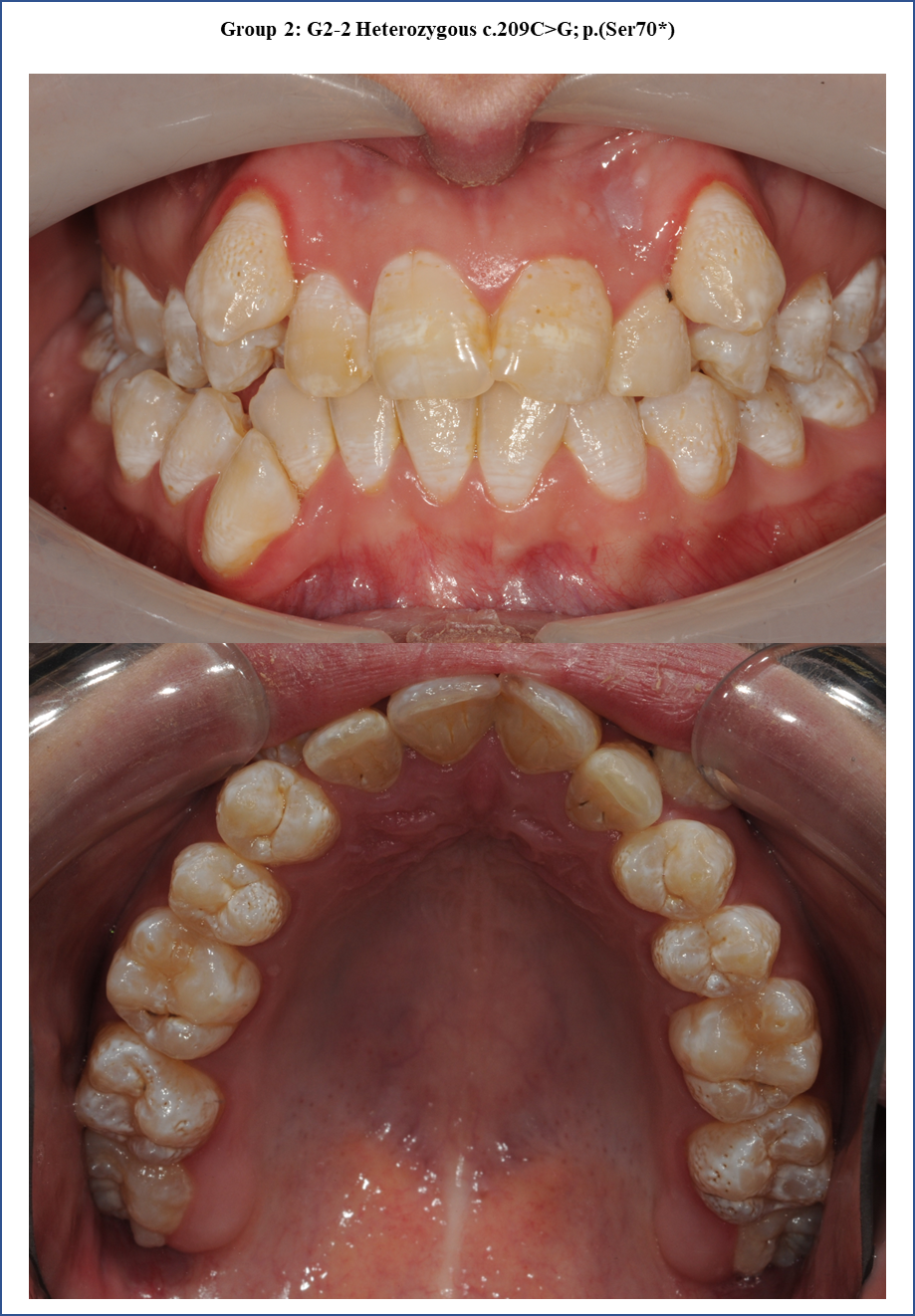


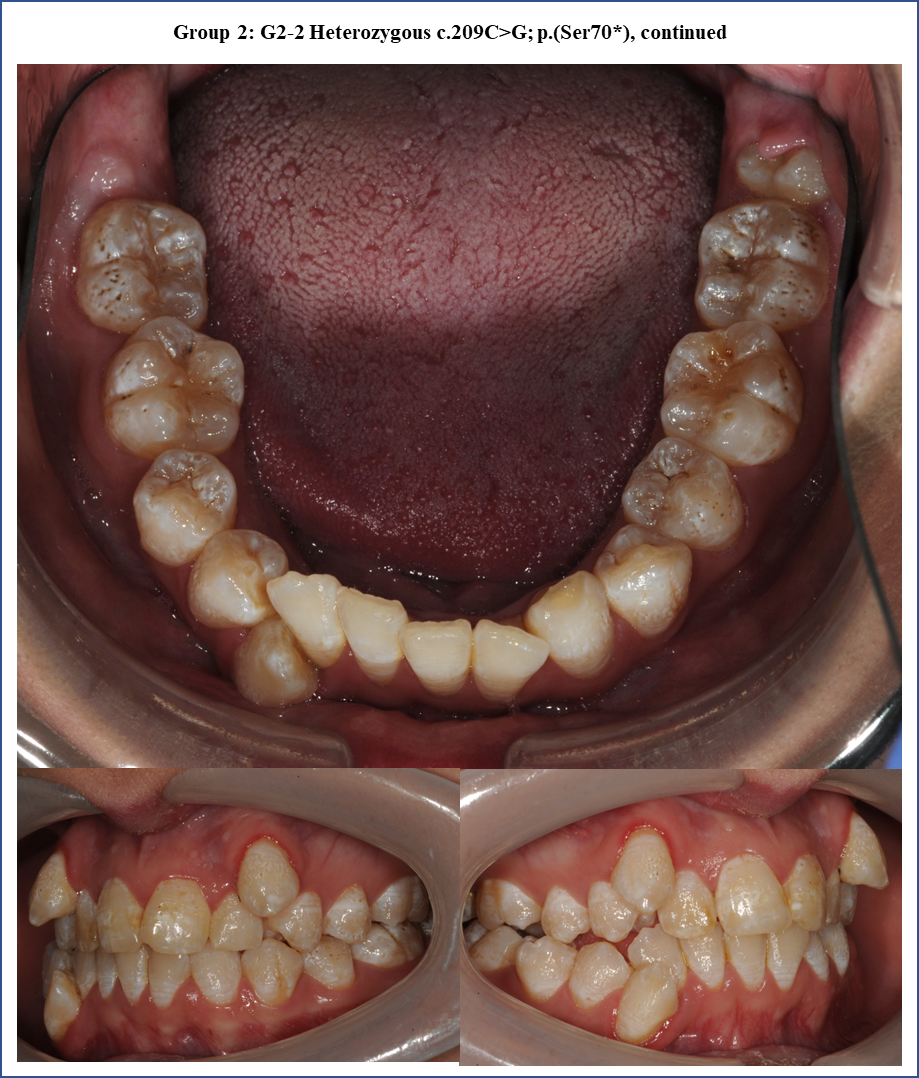


**Images and radiographs from group 3 families
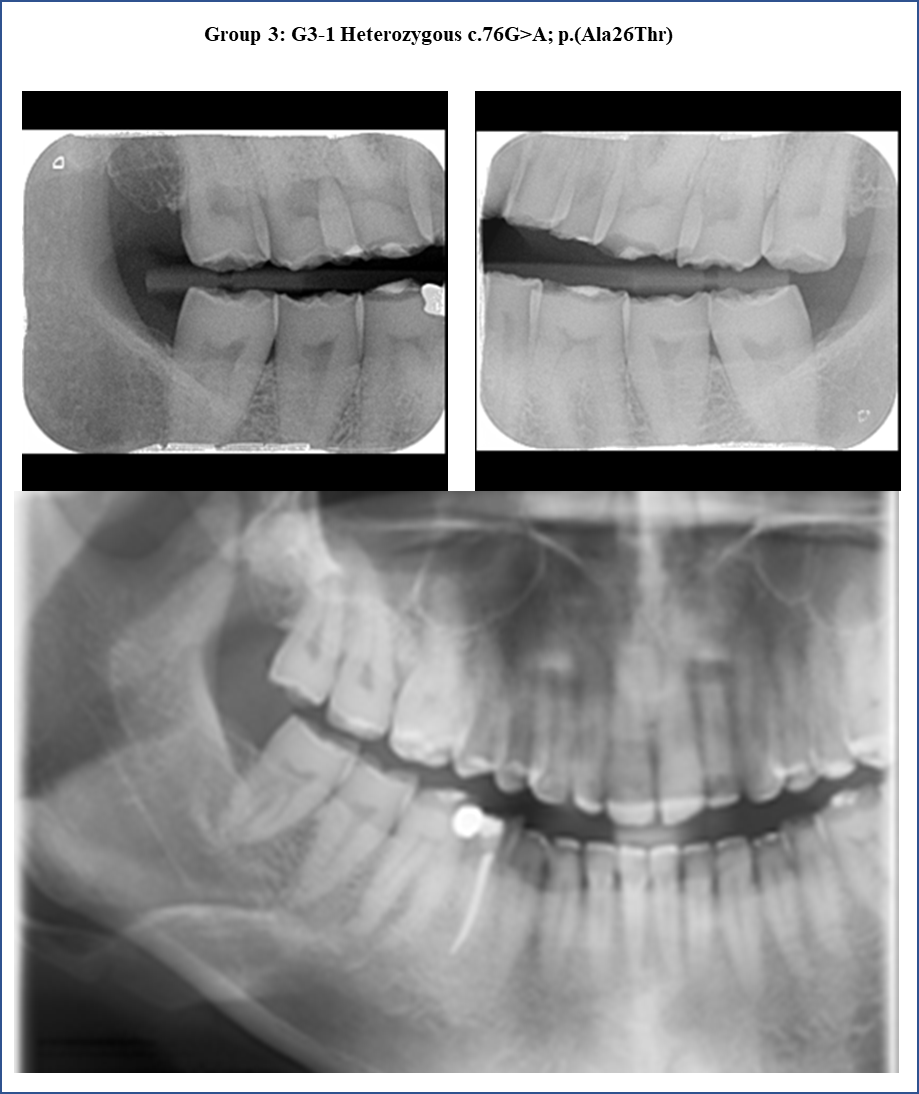
**

Group 3 (G3-1, G3-3) families are characterized by white hypoplastic AI reflecting the presence of a thin layer of enamel on dental radiography.


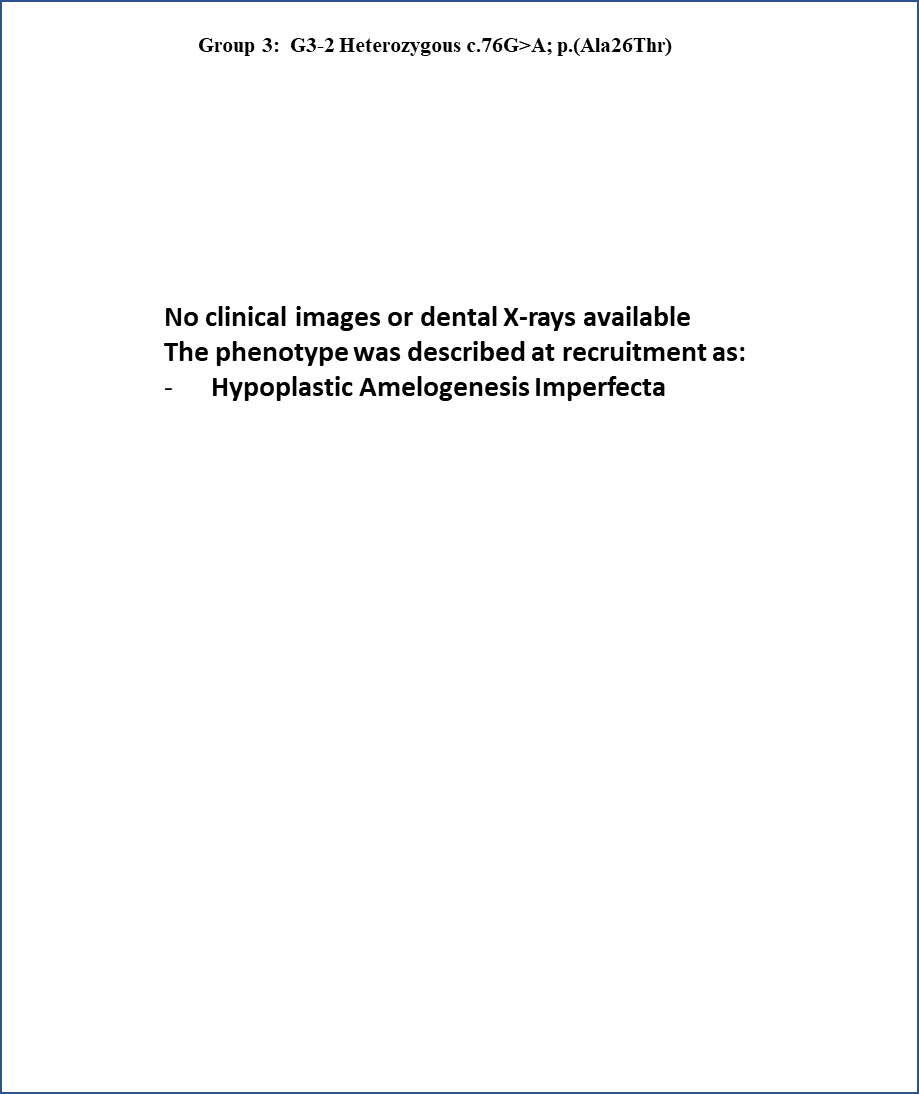


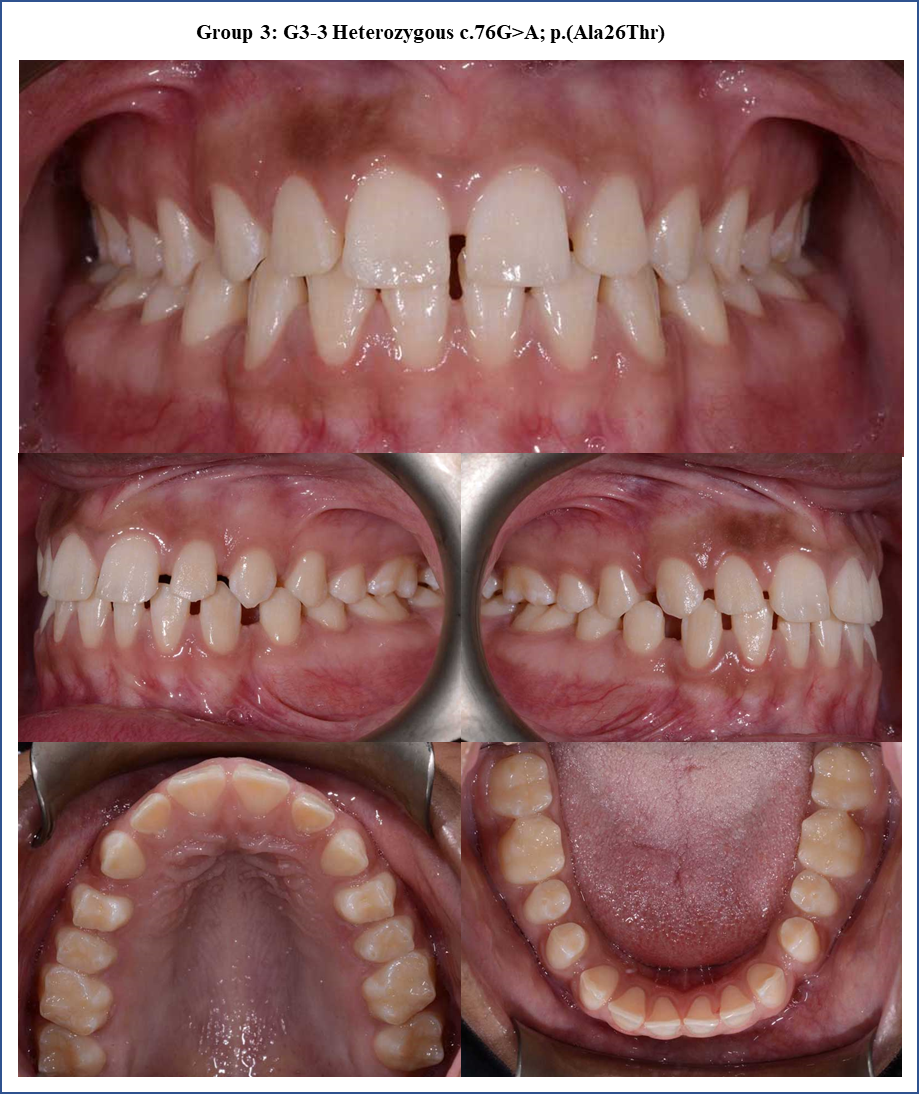


**Figure S2:** Full-gene sequencing of *AMBN* following identification of the heterozygous p.(Ser70*) variant in G2-1 and G2-2. The data displays “full-gene screen” by two amplicons (18201 bp), the p.(Ser70*) is located roughly in the middle of the gene. The identified variants are on the “correct” haplotype in the overlapping area between amplicon 1 and 2 in both G2-1 and G2-2. Haplotype 1 is C defined and haplotype 2 is G defined. The haplotypes were set based on the reference and non-reference nucleotides that define the p.Ser70* mutation.


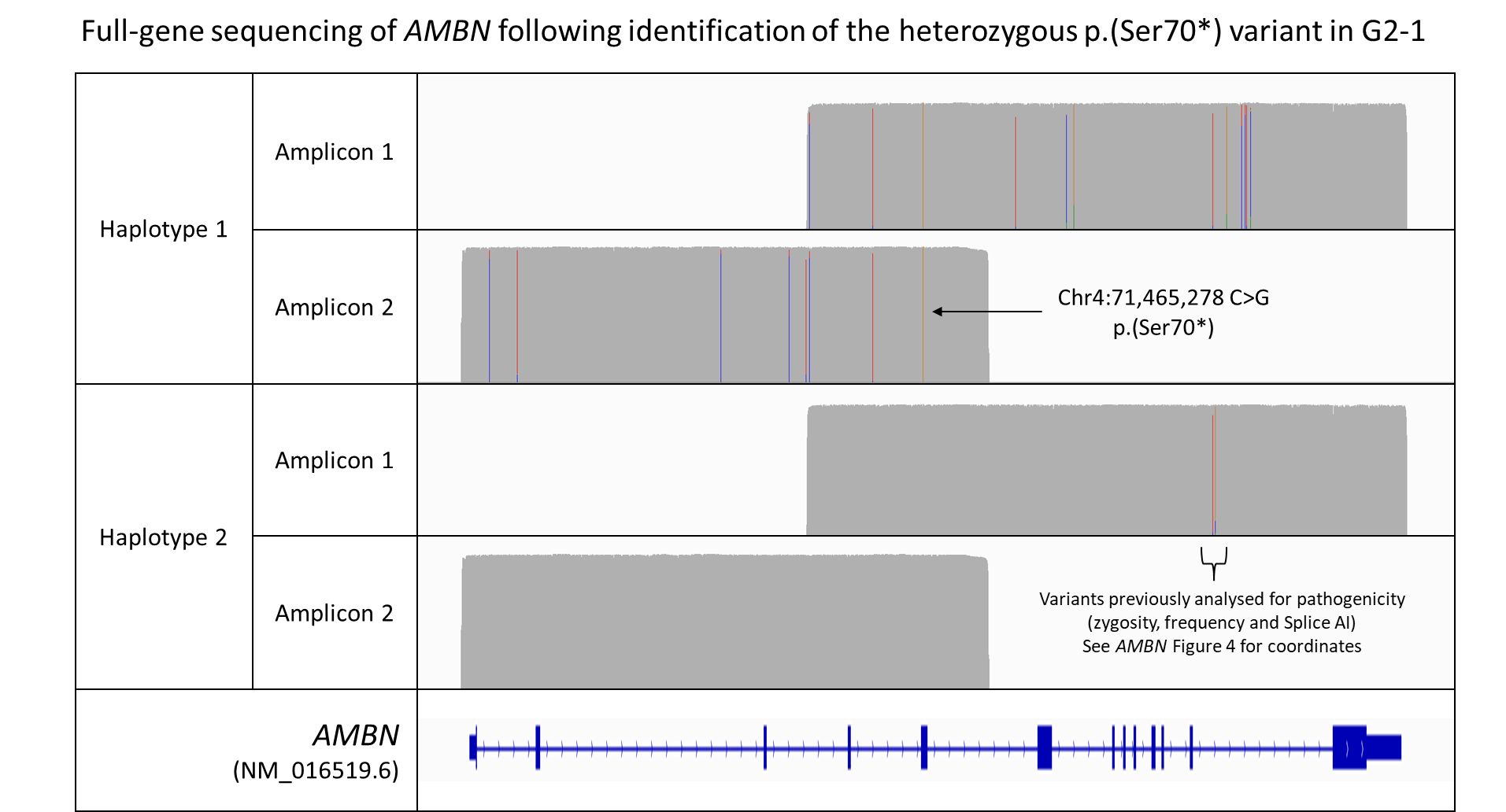


The image displays a 18201bp-long PCR product covering the whole AMBN gene in G2-1. No potential second pathogenic variants or large structural variants were detected other than p.(Ser70*) in the whole gene.


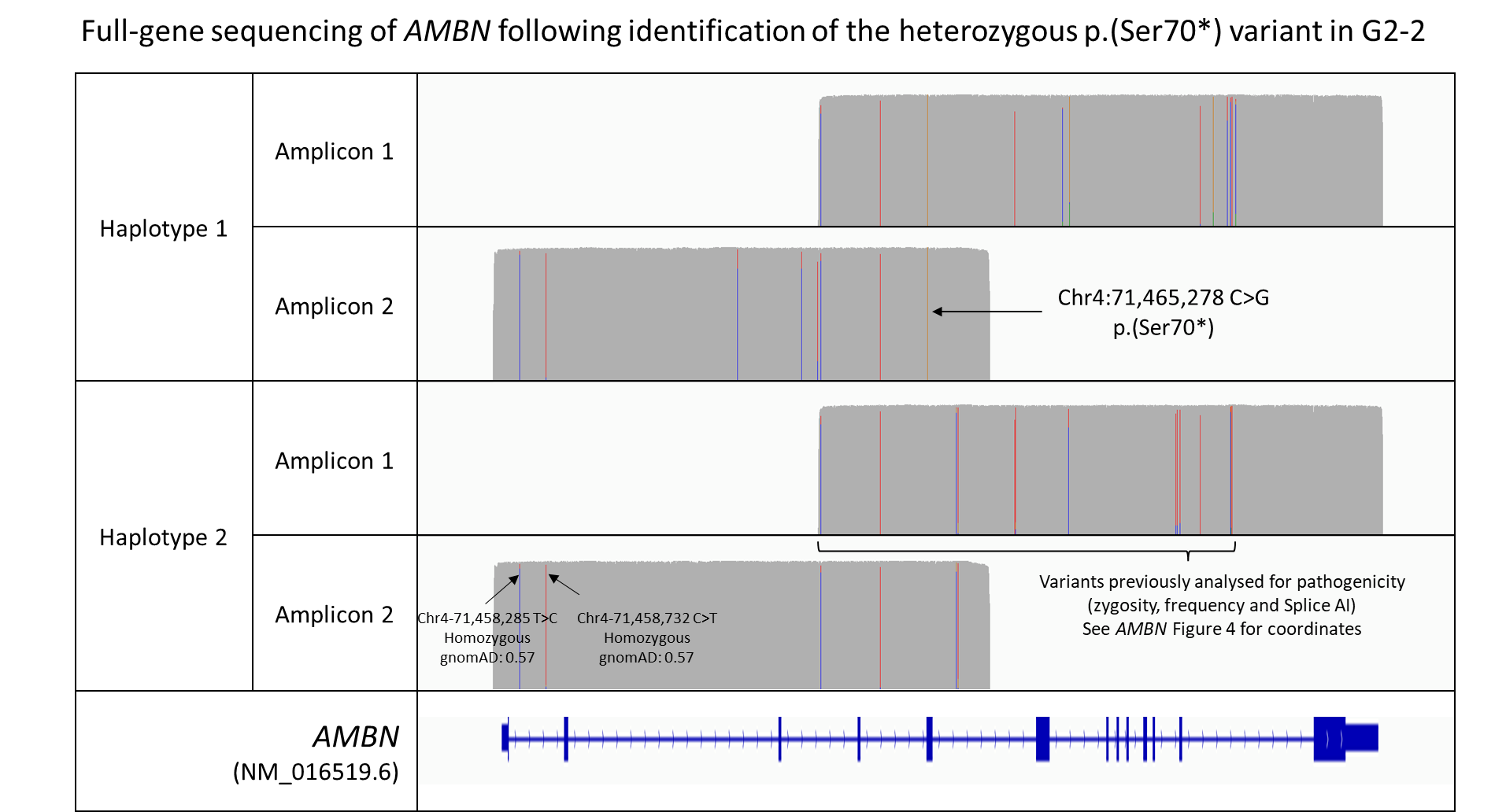


The image displays a 18201bp long PCR product covering the whole AMBN gene in G2-2. No potential second pathogenic variants or large structural variants were detected other than p.(Ser70*) in the whole gene.

1. **Supplementary Table**

**Table S1:** Genes included in the smMIP reagent. Reference genome GRCh37/hg19.

| Gene Name | Gene Symbol | OMIM | Genomic Coordinates | Cytoband |
| --- | --- | --- | --- | --- |
| LAMININ, BETA-3 | *LAMB3* | 150310 | chr1:209,788,218-209,825,820 | 1q32.2 |
| INTEGRIN, BETA-6 | *ITGB6* | 147558 | chr2:160,958,233-161,056,589 | 2q24.2 |
| AMELOTIN | *AMTN* | 610912 | chr4:71,384,298-71,398,459 | 4q13.3 |
| AMELOBLASTIN | *AMBN* | 601259 | chr4:71,457,975-71,473,004 | 4q13.3 |
| ENAMELIN | *ENAM* | 606585 | chr4:71,494,461-71,512,536 | 4q13.3 |
| ODONTOGENESIS-ASSOCIATED PHOSPHOPROTEIN | *ODAPH* | 614829 | chr4:76,481,258-76,491,103 | 4q21.1 |
| FAMILY WITH SEQUENCE SIMILARITY 83 | *FAM83H* | 611927 | chr8:144,806,103-144,815,914 | 8q24.3 |
| COLLAGEN, TYPE XVII, ALPHA-1 | *COL17A1* | 113811 | chr10:105,791,046-105,845,638 | 10q25.1 |
| RECEPTOR EXPRESSED IN LYMPHOID TISSUES | *RELT* | 611211 | chr11:73,087,405-73,108,519 | 11q13.4 |
| MATRIX METALLOPROTEINASE 20 | *MMP20* | 604629 | chr11:102,447,566-102,496,063 | 11q22.2 |
| G PROTEIN-COUPLED RECEPTOR 68 | *GPR68* | 601404 | chr14:91,698,876-91,710,852 | 14q32.11 |
| SOLUTE CARRIER FAMILY 24 (SODIUM/POTASSIUM/CALCIUM EXCHANGER), MEMBER 4 | *SLC24A4* | 609840 | chr14:92,790,152-92,967,825 | 14q32.12 |
| WD REPEAT-CONTAINING PROTEIN 72 | *WDR72* | 613214 | chr15:53,805,938-54,051,859 | 15q21.3 |
| TRANSCRIPTION FACTOR Sp6 | *SP6* | 608613 | chr17:45,922,280-45,928,516 | 17q21.32 |
| DISTAL-LESS HOMEOBOX 3 | *DLX3* | 600525 | chr17:48,067,369-48,072,588 | 17q21.33 |
| FAMILY WITH SEQUENCE SIMILARITY 20, MEMBER A | *FAM20A* | 611062 | chr17:66,531,257-66,597,095 | 17q24.2 |
| ACID PHOSPHATASE 4 | *ACP4* | 606362 | chr19:51,293,672-51,298,481 | 19q13.33 |
| KALLIKREIN-RELATED PEPTIDASE 4 | *KLK4* | 603767 | chr19:51,409,608-51,413,994 | 19q13.41 |
| AMELOGENIN | *AMELX* | 300391 | chrX:11,311,533-11,318,881 | Xp22.2 |

1. **Supplementary references**

Boyle EA, O'Roak BJ, Martin BK, Kumar A, Shendure J. 2014. Mipgen: Optimized modeling and design of molecular inversion probes for targeted resequencing. Bioinformatics. 30(18):2670-2672.

DePristo MA, Banks E, Poplin R, Garimella KV, Maguire JR, Hartl C, Philippakis AA, del Angel G, Rivas MA, Hanna M et al. 2011. A framework for variation discovery and genotyping using next-generation DNA sequencing data. Nat Genet. 43(5):491-498.

Jaganathan K, Kyriazopoulou Panagiotopoulou S, McRae JF, Darbandi SF, Knowles D, Li YI, Kosmicki JA, Arbelaez J, Cui W, Schwartz GB et al. 2019. Predicting splicing from primary sequence with deep learning. Cell. 176(Diaz et al.):535-548.e524.

Karczewski KJ, Francioli LC, Tiao G, Cummings BB, Alföldi J, Wang Q, Collins RL, Laricchia KM, Ganna A, Birnbaum DP et al. 2020. The mutational constraint spectrum quantified from variation in 141,456 humans. Nature. 581(7809):434-443.

Li H, Durbin R. 2009. Fast and accurate short read alignment with burrows-wheeler transform. Bioinformatics. 25(14):1754-1760.

McLaren W, Gil L, Hunt SE, Riat HS, Ritchie GR, Thormann A, Flicek P, Cunningham F. 2016. The ensembl variant effect predictor. Genome Biol. 17(1):122.

Richards S, Aziz N, Bale S, Bick D, Das S, Gastier-Foster J, Grody WW, Hegde M, Lyon E, Spector E et al. 2015. Standards and guidelines for the interpretation of sequence variants: A joint consensus recommendation of the american college of medical genetics and genomics and the association for molecular pathology. Genet Med. 17(5):405-424.

Robinson JT, Thorvaldsdóttir H, Winckler W, Guttman M, Lander ES, Getz G, Mesirov JP. 2011. Integrative genomics viewer. Nat Biotechnol. 29(1):24-26.
