# Supplementary figures and images for: Novel Ameloblastin Variants, Contrasting Amelogenesis Imperfecta Phenotypes
Source: J Dent Res. 2023 Dec 6;103(1):22–30. doi: 10.1177/00220345231203694 (PMC10734210; doi:10.1177/00220345231203694)

## Slide 1
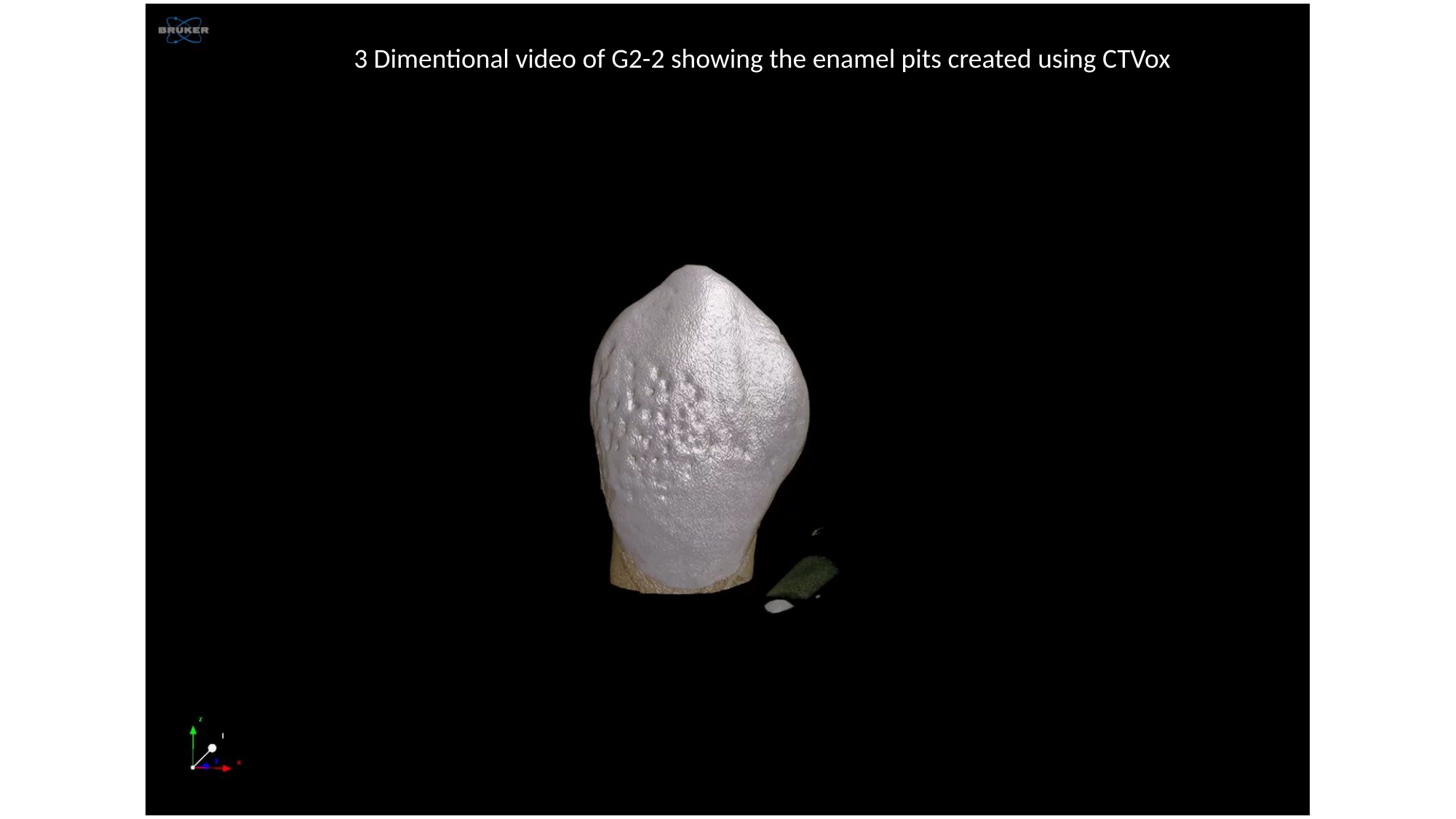

3 Dimentional video of G2-2 showing the enamel pits created using CTVox

Supplement: sj-pptx-1-jdr-10.1177_00220345231203694 – Supplemental material for Novel Ameloblastin Variants, Contrasting Amelogenesis Imperfecta Phenotypes [file sj-pptx-1-jdr-10.1177_00220345231203694.pptx]
